# Pre-pregnancy serum complement C3 level is a predictor of preterm birth for pregnancies with systemic lupus erythematosus

**DOI:** 10.1186/s13075-021-02522-x

**Published:** 2021-05-12

**Authors:** Yuri Hiramatsu, Kentaro Isoda, Takuya Kotani, Eri Nakamura, Yumiko Wada, Youhei Fujiki, Shigeki Makino, Daisuke Fujita, Tohru Takeuchi

**Affiliations:** 1 Department of Internal Medicine (IV), Osaka Medical College, Daigaku-Machi 2-7, Takatsuki, Osaka, 569-8686 Japan; 2 Department of Rheumatology and Allergology, National Hospital Organization Osaka Minami Medical Center, Osaka, Japan; 3 Department of Internal Medicine, Yodogawa Christian Hospital, Osaka, Japan; 4 Department of Internal Medicine, Osaka Medical College Mishima-Minami Hospital, Osaka, Japan; 5 Department of Obstetrics and Gynecology, Osaka Medical College, Osaka, Japan

**Keywords:** Systemic lupus erythematosus, Pregnancy, Preconception care, Preterm birth, Serum complement 3 level

## Abstract

**Background:**

This study aimed to clarify predictors of preterm birth in pregnancy of women with systemic lupus erythematosus (SLE). We investigated the predictors of preterm birth before pregnancy from the perspective of the importance of preconception care.

**Methods:**

We analysed fetal outcomes of 108 pregnancies in 74 SLE patients in a retrospective study. We compared pre-pregnancy clinical characteristics and disease activity in these women between the preterm birth and full-term birth groups to select predictive factors for preterm birth before pregnancy.

**Results:**

Eighty-three of 108 pregnancies resulted in live births, of which 27 (25.0%) were preterm births. Pre-pregnancy serum complement 3 (C3) level was significantly lower in the preterm birth group (77.0 mg/dl) than the full-term birth group (87.5 mg/dl) (*P* = 0.029). Multivariate analysis identified history of lupus nephritis (odds ratio: 5.734, 95% CI 1.568–21.010, *P* = 0.008) and low C3 level (< 85 mg/dl) at pre-pregnancy (odds ratio 4.498, 95% CI 1.296–15.616, *P* = 0.018) as risk factors for preterm birth. The greater the number of these risk factors, the higher was the preterm birth rate (*P* = 0.0007). In the case of SLEDAI score ≤ 4, the preterm birth rate was higher in the pre-pregnancy low C3 group (< 85 mg/dl) (42.1%) than in the high C3 group (C3 ≥ 85 mg/dl) (14.7%) (*P* = 0.018).

**Conclusion:**

For patients with a history of LN, treatment management focusing on pre-pregnancy serum complement levels is very important.

## Background

Systemic lupus erythematosus (SLE) occurs primarily in women of reproductive age [1]. Recent improvements in treatment have dramatically improved the prognosis and quality of life of SLE patients, thus increasing their chances of becoming pregnant and giving birth. In addition, SLE patients are increasingly experiencing pregnancy and childbirth during the course of their treatment. Pregnant patients with SLE are at high risk of developing obstetric complications such as pre-eclampsia and eclampsia, as well as relapse of SLE disease activity during their pregnancy and puerperium.

Furthermore, pregnant patients with SLE have poor birth outcomes such as miscarriage, preterm birth, and fetal growth restriction, and there are many cases of preterm birth even if the pregnancy is successful [2–5]. Increased SLE disease activity during pregnancy, low serum levels of complement 3 (C3) or 4, high oral glucocorticoids (GC) dose, and high SLE disease activity have been reported as factors affecting preterm birth in pregnancy complicated by SLE [6, 7]. Recently, the importance of preconception care has been advocated to reduce these risks that can adversely affect pregnancy outcomes. Preconception care, which is planned management that prepares a women’s body for future pregnancies from the perspective of improving the prognosis for the child, reducing the risk of complications, and maintaining maternal safety, is very important, especially for women with underlying illnesses [3, 8]. However, there are few detailed reports about pre-pregnancy management in women with SLE, even though complications of lupus nephritis and high pre-pregnancy disease activity are known as predictors of preterm birth. Preconception care for pregnancies with SLE has not yet been established [6, 7].

We investigated the factors that influence preterm birth in pregnancies complicated by SLE using a retrospective case-control study method. We report on the predictors of preterm birth before pregnancy, especially in terms of the importance of preconception care.

## Materials and methods

### Patients and study design

The subjects were pregnant SLE patients who were admitted to Osaka Medical College Hospital between January 2000 and October 2020. All patients were managed in our hospital from pre-pregnancy to delivery, and all data were also collected in our hospital.

All of these patients fulfilled the 1997 American College of Rheumatology revised criteria for SLE [9]. Rheumatologists and obstetricians assessed and managed the conditions of each mother and fetus once a month during pregnancy throughout the puerperal period. To treat the patients and perform the present clinical study, past medical histories were investigated from the patients’ previous medical records, and clinical and serological evaluations were performed.

In this study, we measured and evaluated C3 for serum complement levels. Because most complete deficiencies of C4 are associated with SLE patients, in a certain number of patients, serum C4 levels may not correlate with SLE disease activity [10]. Also, CH50 can be difficult to evaluate due to the potential for complement cold-dependent activation [11]. For systemic evaluation, we evaluated serum C3 level and anti-dsDNA antibodies titres every month. The methods used to measure serum C3 levels were liposomal immunoassay before 2019 and turbidimetric immunoassay after 2019. Although the measurement method changed, the reference values were the same and there was no need to correct the serum C3 levels. Antinuclear antibodies, anti-dsDNA antibodies, anti-RO/SSA and anti-RNP antibodies, and antiphospholipid antibody (aPL) (i.e., against cardiolipin or b2-glycoproteins by ELISA or lupus anticoagulants) were also evaluated. For lupus nephritis, we determined that cases for which a definitive diagnosis could not be obtained by renal biopsy were nephritis based on the presence of proteinuria (0.5 g/day or higher) or cellular casts. If an increase in urinary protein level or blood pressure was observed during pregnancy, we judged this to indicate a flare of lupus nephritis based on a history of lupus nephritis, hypocomplementaemia, and an increase in anti-DS-DNA antibody titre or SLEDAI score.

This study was conducted in accordance with the Declaration of Helsinki and its amendments and was approved by the Osaka Medical College, Faculty of Medicine Ethics Committee (approval no. 1526). Written informed consent was obtained from each patient.

### Treatment for SLE

We investigated GC and immunosuppressants such as azathioprine (AZA), tacrolimus (TAC), and hydroxychloroquine (HCQ) with regard to treatment contents before and during pregnancy. We also examined past use of GC pulse therapy and intravenous cyclophosphamide (IVCY). GC and immunosuppressants in use before pregnancy were continued during pregnancy. Previously, we treated SLE only with GC from pre-pregnancy to after delivery, and all patients continued to use GC even after their pregnancy was confirmed. In recent years, since the use of immunosuppressants during pregnancy has been permitted, all patients have been treated with immunosuppressants drugs before pregnancy and have continued to be treated with them even after their pregnancy is confirmed. In the patients with SLE flare during pregnancy and postpartum, GC or immunosuppressants were newly administered and/or increased. Low-dose aspirin (LDA) was administered to patients positive for antiphospholipid antibody, and unfractionated heparin (UFH) was also used in addition to LDA in the patients with severe antiphospholipid antibody syndrome (APS), those who were positive twice or more for lupus anticoagulant, or those with a frequent history of obstetric complications.

### Evaluation of fetal outcome and disease activity of SLE

Preterm birth was defined as delivery after 28 weeks but earlier than 37 weeks gestation (including termination of pregnancy at < 37 weeks due to hypertensive disorders of pregnancy, preeclampsia or SLE flare) Spontaneous abortion was defined as completion of pregnancy earlier than 22 weeks of gestation. Stillbirth was defined as fetal death at 28 weeks of gestation or later. Abortion was performed at the patient’s own request. We also evaluated the relapse and development of major organ lesions of SLE. In this study, based on the Asia Pacific League of Associations for Rheumatology LLDAS (lupus low disease activity state), the condition in which all of the following four items were satisfied was defined as “SLE disease activity: less than low disease activity”: (1) SLEDAI-2K (Systemic Lupus Erythematosus Disease Activity Index 2000) ≤4, with no activity in major organ systems (renal, central nervous system, cardiopulmonary, vasculitis, and fever) and no hemolytic anaemia or gastrointestinal activity; (2) no new features of lupus disease activity compared with the previous assessment; (3) Physician Global Assessment (PhGA) (0–3) ≤1; and (4) well-tolerated standard maintenance dosages of immunosuppressive drugs and approved biologics [12]. In this study, we excluded GC use from the disease activity endpoint as we are targeting pregnant patients in whom the combination of immunosuppressive drugs was restricted, and the oral dose of GC was relatively high. Flares were defined using the SELENA-SLEDAI Flare Index. Relapse of SLE disease activity was defined as an increase in the SLEDAI score to ≥ 3 and exacerbation of organ damage [13]. The activity of lupus nephritis was evaluated by proteinuria of 0.5 g/day or more, an increase in serum Cr level, and an increase in blood pressure.

### Statistical analysis

Data are presented as the median value (interquartile range [IQR]) or number of subjects. Statistical analyses were performed with the chi-squared test or Fisher’s exact test for binary data and with the Mann–Whitney *U* test for continuous data between two groups. From these analyses, we selected candidates as the predictors of premature birth and then performed receiver operating characteristic (ROC) curve analysis to determine the most suitable cut-off level. A multivariate logistic regression model was used, and odds ratio (OR) and 95% confidence interval (CI) were calculated to examine independent risk factors for premature birth. A *P* value of < 0.05 was considered significant. Statistical calculations were performed using the statistical software JMP Pro for Windows, version 13.0 (SAS Institute Inc., Cary, NC, USA).

## Results

### Patient characteristics of pre-pregnancy

In total, 108 pregnancies in 74 patients were analysed, and the patients’ characteristics are shown in Table 1. Of these 74 patients, 48 became pregnant once, 21 twice, three patients three times, one patient four times, and two patients 5 times during the study period. Patient age at pregnancy was 33 (30–36) years. The SLEDAI score was 0 (0–4). However, the SLEDAI score was missing in two patients because the items to be evaluated were insufficiently described in their previous medical records.

**Table 1:** Fetal outcome and patient characteristics and medications prior to pregnancy

	*n* = 108
Fetal outcome	
Live birth, *n* (%)	83 (76.9)
Full-term birth, *n* (%)	56 (51.9)
Preterm birth, *n* (%)	27 (25.0)
Miscarriage, *n* (%)	11 (10.2)
Fetal loss, *n* (%)	3 (2.8)
Elective abortion, *n* (%)	11 (10.2)
Age, years old	33 (30 - 36)
SLE duration, years	8 (4 - 13)
History of lupus nephritis, *n* (%)	29 (26.9)
SLEDAI, score	0 (0 - 4)
Past use of steroid pulse therapy, *n* (%)	27 (25.0)
Past use of IVCY, *n* (%)	14 (13.0)
Prednisolone, *n* (%)	91 (84.3)
Prednisolone dose, mg/day	10 (5 - 12)
Azathioprine, *n* (%)	23 (21.3)
Tacrolimus, *n* (%)	49 (45.3)
HCQ, *n* (%)	11 (10.2)
Serum C3 level, mg/dL	81 (74 - 92)
Positive Anti-ds-DNA Ab, *n* (%)	73 (67.6)
Anti-dsDNA Ab, IU/ml	5.1 (2.5 - 8.4)
Positive anti-Ro/SS-A Ab, *n* (%)	38 (35.2)
Positive LA or anti-aCL-IgG Ab or anti-β2GP1 Ab	46 (43)
Positive anti-RNP Ab, *n* (%)	35 (32.4)
Positive anti-Sm Ab, *n* (%)	9 (8.3)

Data are presented as median value (interquartile range) or number of patients

Laboratory markers were evaluated in all patients. *SLE* systemic lupus erythematosus, *SLEDAI* SLE Disease Activity Index, *IVCY* intravenous cyclophosphamide therapy, *HCQ* hydroxychloroquine, *C3* complement 3, *anti-dsDNA Ab* anti-double-stranded DNA antibodies, *anti-Ro/SS-Ab* anti-Ro/SSA antibodies, *LA* lupus anticoagulant, *aCL* anticardiolipin, *Β2Gp1* anti-β2-glycoprotein 1

One patient was complicated with active lupus nephritis, but there were no active major organ lesions in the other patients. In the past, 29 patients (26.9%) were diagnosed as having lupus nephritis. The serum C3 level before pregnancy was 81 (74–92) mg/dl. Anti-dsDNA antibody and anti-RO/SS-A antibody were positive in 73 (67.6%) and 38 patients (35.2%), respectively. Forty-six patients (43%) were also positive for lupus anticoagulant activity, anti-cardiolipin IgG antibody, or anti-β-2GP1 antibody. Fifteen patients were complicated by APS and 38 by Sjögren’s syndrome. Ninety-one patients (84.3%) were treated with 10 (5–12) mg/dl of GC before pregnancy. GC pulse therapy was previously administered in 27 patients (25.0%). LDA was administered to 15 aPL-positive patients with a history of obstetrical abnormalities, and UFH was co-administered from the first trimester.

### Maternal and fetal outcome

**Fig. 1:**
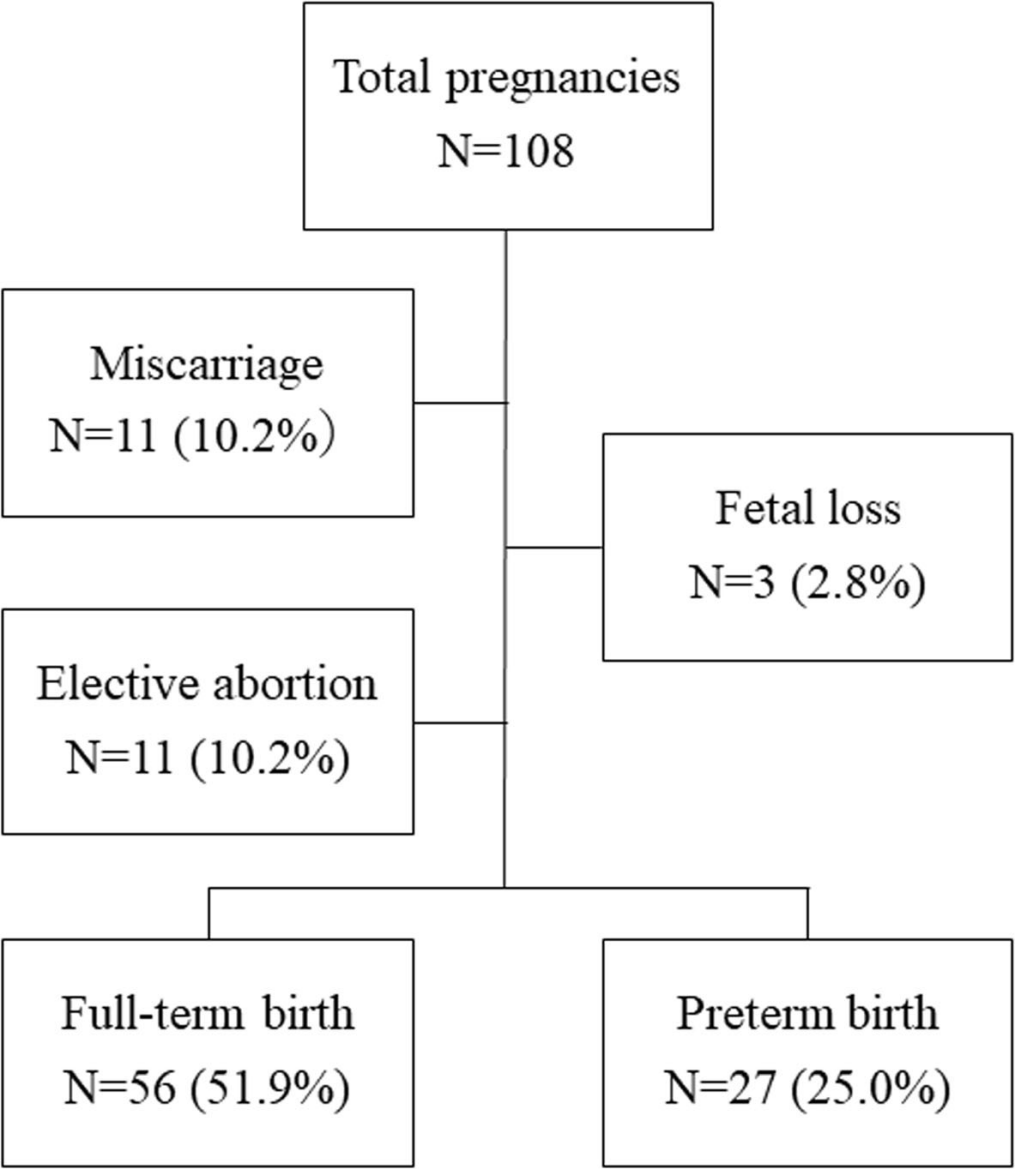
The fetal outcomes of all 108 pregnancies. Eighty-three pregnancies (76.9%) resulted in live births, 11 in miscarriage (at 5–11 weeks), 3 in stillbirth, and 11 in abortion. Twenty-seven of the 82 live births were preterm birth

Figure 1 shows the fetal outcomes of all 108 pregnancies. Eighty-three pregnancies (76.9%) resulted in live births, 11 in miscarriage (at 5–11 weeks), 3 in stillbirth, and 11 in abortion. Two patients with APS experienced stillbirth at 22 and 25 weeks, respectively, and one patient had chromosome aberration at 15 weeks. The reasons for abortion were social background, poor control of the underlying disease, and taking medications that are contraindicated during pregnancy. The gestational age at delivery was 37.0 (33.5–37) weeks, and the fetal birth weight was 2760 (2289–3007) g. Twenty-seven (32.5%) of the 82 live births were preterm birth, and 2 were terminated before 30 weeks of gestation due to maternal preeclampsia. Lupus nephritis relapsed in two patients, and their pregnancies ended before 32 weeks. Fifty women (60.2%) delivered spontaneously, and 33 woman (39.7%) delivered by caesarean section. Eleven (13.2%) patients developed gestational hypertension, 8 (9.6%) preeclampsia, and 8 (9.6%) gestational diabetes.

### Comparison of patient backgrounds between preterm and full-term birth groups

Table 2 shows the pre-pregnancy clinical backgrounds of the preterm and full-term birth groups. There was no difference in age, duration of illness, or SLEDAI score between the two groups. The preterm birth group had a significantly higher history of lupus nephritis than the full-term birth group (*P* < 0.001). There was no difference in the positive rate and antibody titer of anti-dsDNA antibody, or those in the other autoantibodies, between the two groups. Pre-pregnancy serum C3 levels were significantly lower in the preterm birth group (77.5 mg/dl) than in the full-term birth group (87.5 mg/dl) (*P* = 0.027). In terms of treatment, there was no significant difference in the dose of GC administered before pregnancy, but the past use of GC pulse therapy was higher in the preterm birth group (*P* = 0.009).

**Table 2:** Comparison of patient backgrounds of full-term birth and preterm birth

	Full-term birth	Preterm birth	*p* value
Number of patients	56	27	
Gestational age at delivery, weeks	38.0 (37.0–39.0)	34.0 (32.0–36.0)	< 0.001
Birth weight, g	2892 (2610–3115)	2154 (1568–2400)	< 0.001
Age, years	32 (30–36)	34 (31–36)	0.249
SLE duration, years	7.5 (3.3–11.8)	9.0 (5.0–16.0)	0.056
Lupus nephritis, *n* (%)	8 (14.3)	14 (51.9)	< 0.001
APS, *n* (%)	21 (37.5)	12 (44.4)	0.634
SLEDAI, score	0 (0–4)	1 (0–4)	0.225
SLEDAI ≥ 4, *n* (%)	3 (5.4)	5 (18.5)	0.103
Preeclampsia, *n* (%)	3 (5.4)	5 (18.5)	0.106
Comorbidity			
Diabetes mellitus, *n* (%)	5 (8.9)	6 (22.2)	0.163
Hypertension, *n* (%)	3 (5.4)	5 (18.5)	0.106
Past use of steroid pulse therapy, *n* (%)	7 (12.5)	11 (40.7)	0.009
Past use of IVCY, *n* (%)	6 (10.7)	5 (18.5)	0.326
Prednisolone dose, mg/day	9.0 (3.1–11.0)	10.0 (7.0–12.0)	0.181
Azathioprine, *n* (%)	14 (25.0)	3 (11.1)	0.245
Tacrolimus, *n* (%)	25 (44.6)	15 (55.6)	0.482
HCQ, *n* (%)	5 (8.9)	4 (14.8)	0.463
Serum C3 level, mg/dL	87.5 (75.0–96.3)	77.5 (68.2–84.8)	0.027
Positive Anti-ds-DNA Ab, *n* (%)	35 (62.5)	20 (74.1)	0.333
Anti-dsDNA Ab, IU/ml	5.9 (3.0–8.4)	3.0 (1.8–7.4)	0.082
Positive anti-Ro/SS-A Ab, *n* (%)	19 (33.9)	10 (37.0)	0.810
Positive anti-RNP Ab, *n* (%)	19 (33.9)	9 (33.3)	1.000
Positive anti-Sm Ab, *n* (%)	3 (5.4)	3 (11.1)	0.385

Data are presented as median value (interquartile range) or number of patients. The *p* values were estimated using Fisher’s exact test or Wilcoxon rank sum test

*SLE* systemic lupus erythematosus, *SLEDAI* SLE Disease Activity Index, *APS* antiphospholipid syndrome, *IVCY* intravenous cyclophosphamide therapy, *HCQ* hydroxychloroquine, *C3* complement 3, *Anti-dsDNA Ab* anti-double-stranded DNA antibodies, *anti-Ro/SS-Ab* anti-Ro/SSA antibodies, *anti-RNP Ab* anti-RNP antibodies, *anti-Sm Ab* anti-Sm antibodies

**Fig. 2:**
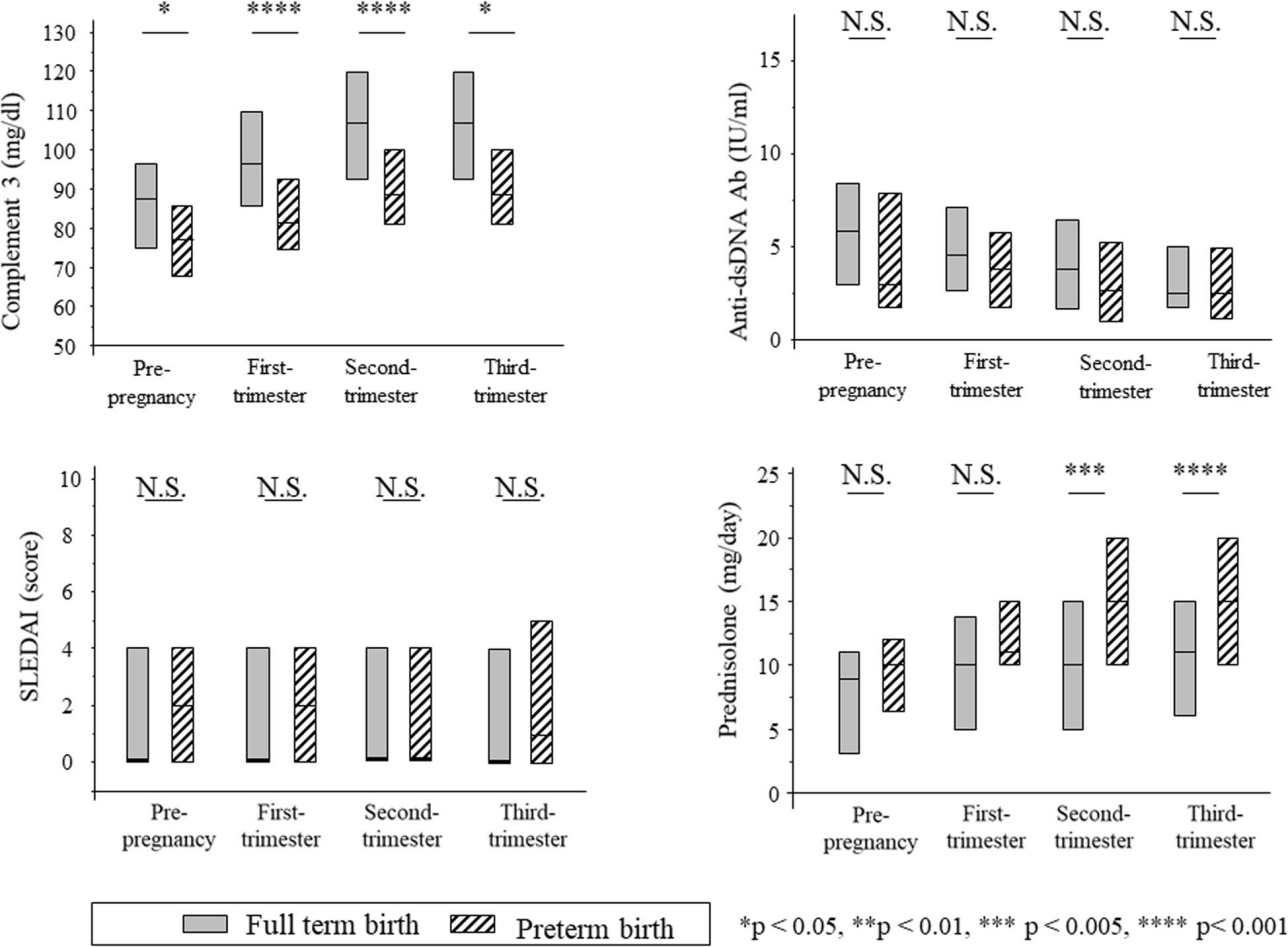
The transition of each parameter before and after pregnancy between the full-term birth group and preterm birth group. **a** Serum C3 levels of the preterm birth group were lower than those of the full-term birth group during all periods. **b** There were no significant differences between in anti-dsDNA antibody titre during all periods. **c** There were no significant differences in SLEDAI score during all periods. **d** The doses of GC in the second and third trimesters were significantly higher in the preterm birth. *P< 0.05, ***P < 0.005, ****P < 0.001, N.S. Not significant

Figure 2 shows the transition of each parameter before and during pregnancy between the full-term birth group and preterm birth group. In the preterm birth group versus full-term birth group, serum C3 levels were lower in the first, second, and third trimesters (81 vs 97 mg/dl, *P* < 0.001; 88 vs 107 mg/dl, *P* < 0.001; and 100 vs 107 mg/dl, *P* = 0.024, respectively). However, there was no significant difference between the two groups in anti-dsDNA antibody titer and SLEDAI score during pregnancy. The dose of GC during pregnancy was significantly higher in the preterm birth group in the second and third trimesters (*P* = 0.01 and *P* = 0.001, respectively).

### Selection of risk factors for preterm birth before pregnancy

We focused on the serum C3 level as a predictor of preterm birth before pregnancy based on a comparison of patient backgrounds in the preterm and full-term birth groups. We performed a ROC analysis to determine a cut-off value for pre-pregnancy serum C3 level that could indicate risks for preterm birth. As a result, the cut-off value for the serum C3 level was set to 84 mg/dl (sensitivity: 0.76, specificity: 0.54, AUC: 0.653, *p* = 0.043). We then performed a multivariate analysis to select risk factors for preterm birth before pregnancy. Candidate risk factors obtained from the results of univariate analysis comparisons between the two groups included age at pregnancy, which is commonly cited as a risk of preterm birth, history of lupus nephritis, past use of GC pulse therapy, and serum C3 level < 85 mg/dl. The multivariate analysis using these variables identified a history of lupus nephritis (OR 6.267, 95% CI 1.750–22.443, *P* = 0.005) and pre-pregnancy serum C3 level < 85 mg/dl (OR 4.754, 95% CI 1.373–16.461, *P* = 0.014) as the risk factors for preterm birth (Table 3). In addition, among the patients with APS, the frequency of preterm birth was significantly higher in the C3 <85 mg/dl group than that in the C3 ≥ 85 mg/dl group (*P* = 0.0347).

**Table 3:** Risk factors of preterm birth

	Odds ratio	95%CI	*p* value
Age at pregnancy (per unit)	1.134	0.977–1.328	0.086
History of lupus nephritis	6.267	1.750–22.443	0.005
Low serum complement 3 (< 85 IU/ml)	4.754	1.373–16.461	0.014
Past use of steroid pulse therapy	2.640	0.708–9.845	0.148

A multivariate logistic regression model. A *p* value of < 0.05 was considered significant

**Fig. 3:**
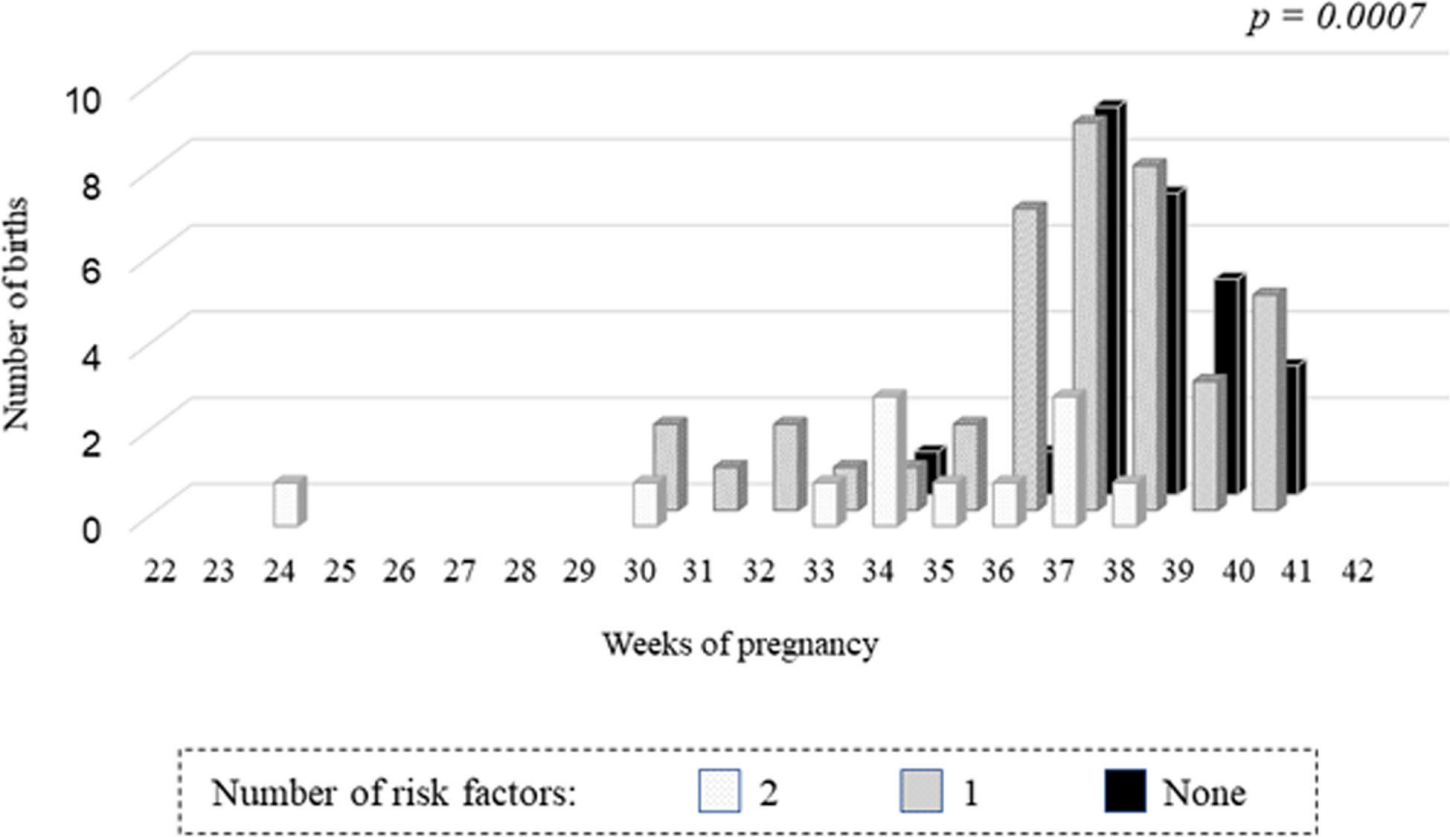
The relation between the number of risk factors and the number of weeks of delivery. The preterm birth rate (preterm birth/total birth ratio) was 7.7% (2/26) in the patients without risk factors (a history of lupus nephritis and pre-pregnancy serum C3 level < 85 mg/dl), 39.0% (16/41) in those with 1 risk factor, and 66.7% (8/12) in those with 2 risk factors. The greater the number of these risk factors, the higher was the preterm birth rate (*P* = 0.0007)

Figure 3 shows the relationship between the number of risk factors (a history of lupus nephritis and pre-pregnancy serum C3 level < 85 mg/dl) and the number of weeks of delivery. The preterm birth rate (preterm birth/total birth ratio) was 7.7% (2/26) in the patients without risk factors, 39.0% (16/41) in those with 1 risk factor, and 66.7% (8/12) in those with 2 risk factors. The greater the number of these risk factors, the higher was the preterm birth rate (*P* = 0.0007).

We compared the serum C3 levels and history of lupus nephritis between the spontaneous delivery group (*n* = 50) and caesarean section group (*n* = 33). There was no difference in serum C3 levels between the two groups. However, the frequency of lupus nephritis history was significantly higher in the caesarean section group than that in the spontaneous delivery group (*p* = 0.042).

### Relationship between the pre-pregnancy serum C3 levels and the number of weeks of delivery in the cases of SLEDAI score ≤ 4

**Fig. 4:**
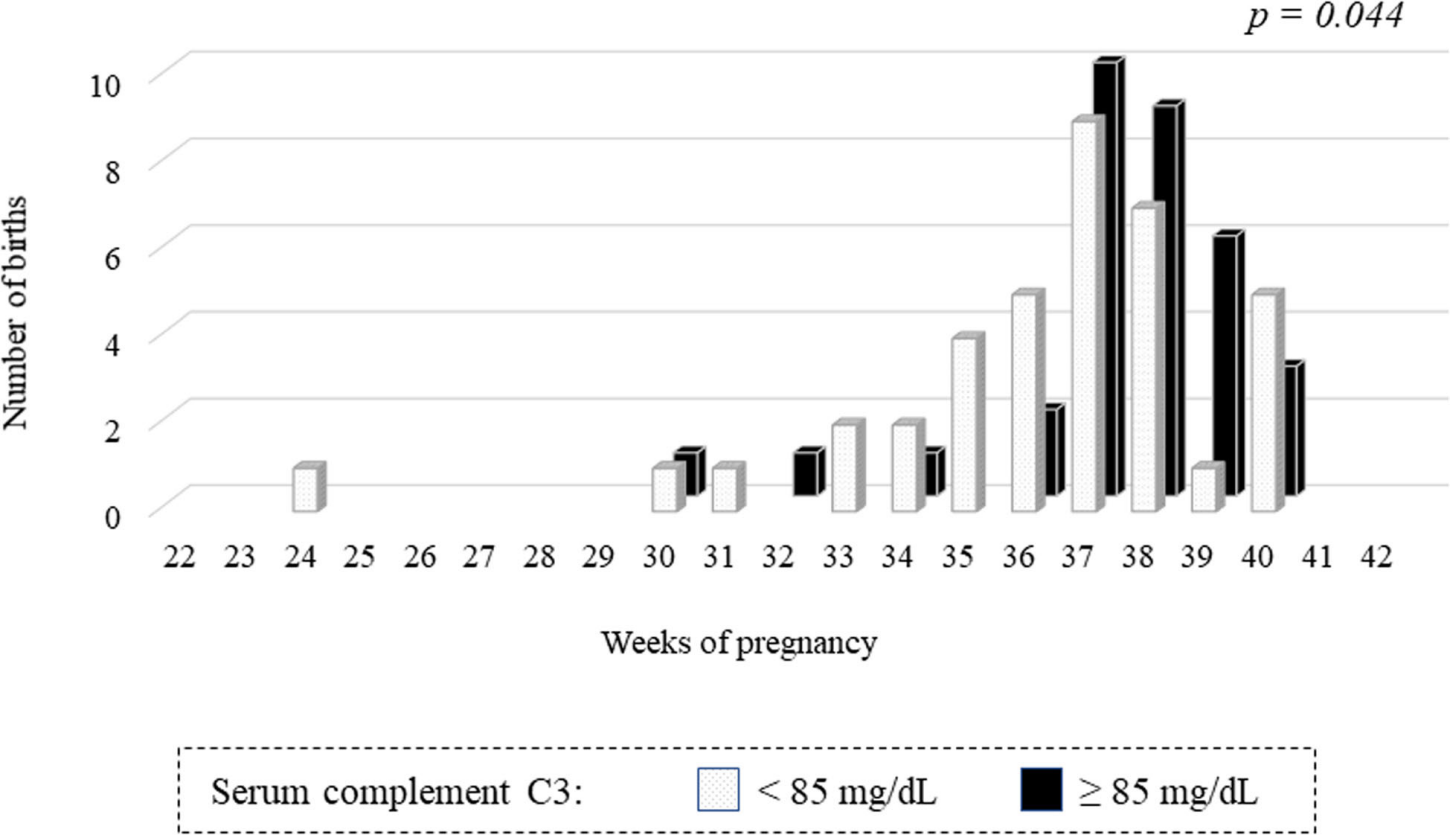
The relationship between the pre-pregnancy serum C3 level and the number of weeks of delivery in the cases of SLEDAI score ≤ 4. The preterm birth rate was significantly higher in the pre-pregnancy low C3 group (< 85 mg / dl) (42.1%) than that in the high C3 group (C3 ≥ 85 mg / dl) (14.7%) (*P* = 0.018). The number of weeks of delivery was 37 (35–38) in the low C3 group, which was significantly earlier than that in the high C3 group 38 (37–39) (*P* = 0.044)

In pregnancies with a SLEDAI score > 4, 5 of 7 (71.4%) had preterm birth, and with a SLEDAI score ≤ 4, 21 of 72 (29.1%) had preterm birth. Figure 4 shows the relationship between the pre-pregnancy serum C3 levels and the number of weeks of delivery in the cases of SLEDAI score ≤ 4. The preterm birth rate was significantly higher in the pre-pregnancy low C3 group (< 85 mg/dl) (42.1%) than that in the high C3 group (≥ 85 mg/dl) (14.7%) (*P* = 0.018). The number of weeks of delivery was 37 (35–38) in the low C3 group, which was significantly earlier than that in the high C3 group 38 (37–39) (*P* = 0.044).

### Maternal disease activity during pre-pregnancy and pregnancy

Among the 83 women delivering live babies, 7 (8.4%) had SLE disease activity (medium disease activity or higher) before pregnancy. The pre-pregnancy serum C3 level was 81 (74–92) mg/dl of all. In addition, the presence of proteinuria or cellular casts of 0.5 g/day or more was observed before pregnancy in one woman.

Twenty women (24.0%) had increased SLE disease activity (increase in the SLEDAI score of > 3) during pregnancy. Of the women who had undergone GC therapy before pregnancy, 43 (51.8%) had an increased level of GC (8.9 mg/day). GC was newly introduced in 4 women (4.8%) after their pregnancy was discovered. Due to SLE relapse during pregnancy, immunosuppressants were newly initiated during pregnancy in 5 women.

**Fig. 5:**
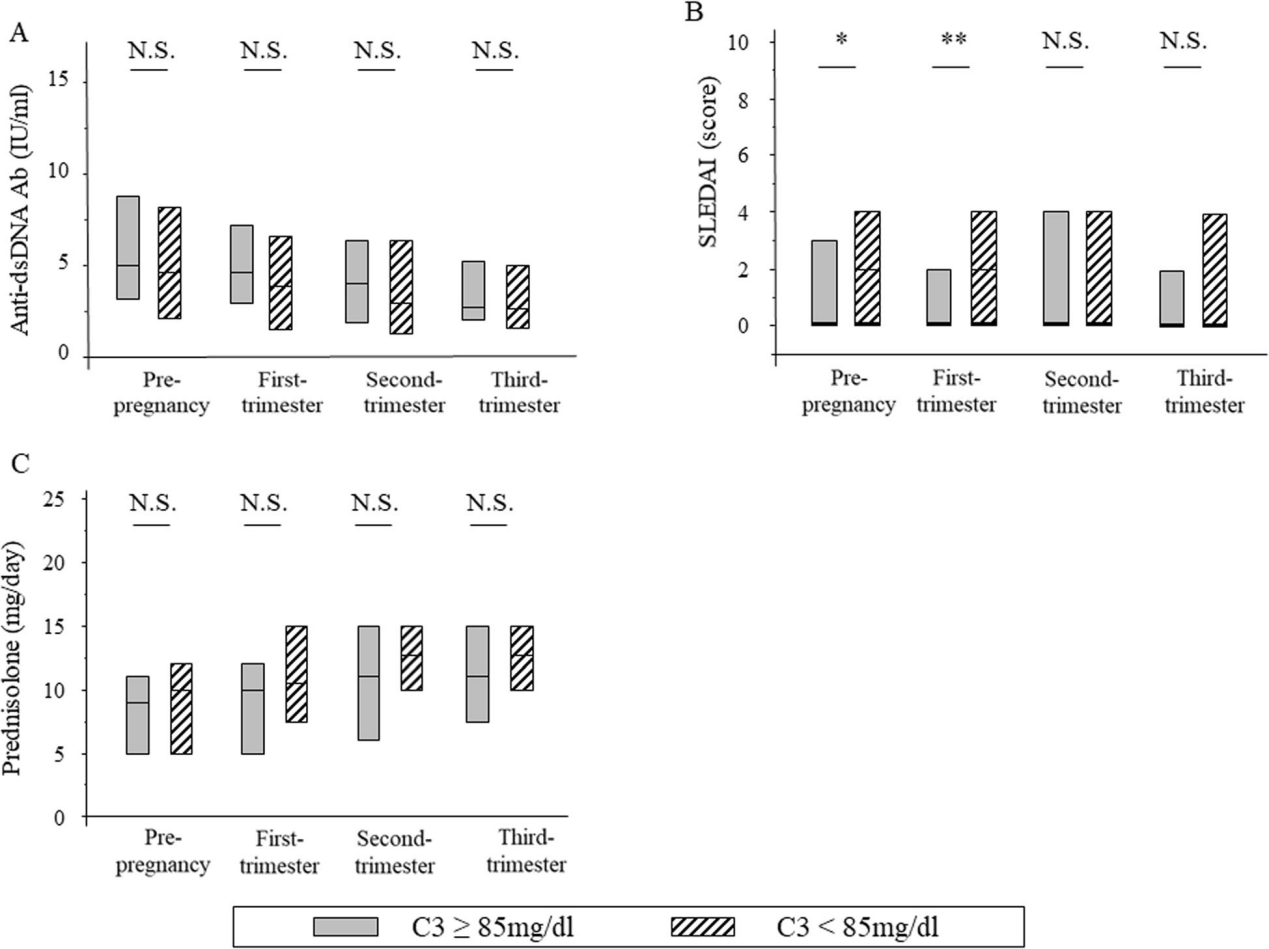
Comparison of each parameter during pregnancy between the high (≥ 85 mg/dl) and low C3 group (< 85 mg/dl) at the time of pre-pregnancy. **a** The titres of anti-dsDNA antibodies were not significantly different during all periods. **b** SLEDAI scores at pre-pregnancy and the first trimester of pregnancy were lower in the high C3 group. **c** The doses of glucocorticoids were not significantly different during all periods. *P < 0.05, **P < 0.01, ****P < 0.001, N.S. Not significant

We compared SLE disease activity between the preterm and full-term birth groups (Fig. 2). There was no significant difference in the SLEDAI score and anti-dsDNA antibody titres during either pre-pregnancy or pregnancy. Figure 5 shows a comparison of each parameter during pregnancy in the two groups [high (≥ 85 mg/dl) and low C3 group (< 85 mg/dl)] divided by serum C3 levels at pre-pregnancy. The SLEDAI score was lower in the high C3 group before and in the first trimester than those in the low C3 group, but no significant correlation was observed in the anti-dsDNA antibody titre in either group during pre-pregnancy pregnancy.

## Discussion

We investigated predictors of preterm birth in a retrospective case-control study from the perspective of preconception care for pregnancies in women with SLE. The preterm birth group had lower serum C3 levels than the full-term birth group before pregnancy, which indicated that a pre-pregnancy serum C3 level < 85 mg/dl and a history of lupus nephritis are potential risk factors for preterm birth in pregnancies with SLE. We also found that the higher the number of these risk factors in pregnant patients with SLE, the higher was the preterm birth rate. Furthermore, it was clarified that the risk of preterm birth increases in cases without SLE disease activity (SLEDAI score ≤ 4), but with low C3 level (< 85mg/dl) before pregnancy. To our knowledge, there have been no previous reports showing a relation between pre-pregnancy serum C3 levels and preterm birth in pregnancies with SLE. This is the first report to show that the combination of pre-pregnancy serum C3 level and a history of lupus nephritis may predict preterm birth in pregnancies with SLE.

Pregnancy in women with SLE carries a higher risk of pregnancy complications than that in healthy individuals, and preterm births are reported to be 2.5 times higher [5]. Preterm birth is divided into spontaneous preterm birth due to premature rupture of the membrane and imminent preterm labour, and artificial preterm birth due to preeclampsia-induced hypertension and fetal adaptation. Preterm birth is the leading cause of neonatal death [14]. It is important to identify the risk factors of preterm births because in the surviving infants, the prevalence of diseases such as chronic lung disease, intraventricular haemorrhage, periventricular leukomalacia, and others is high, and they can affect the prognosis of child development [15]. There are some reports of outcomes in pregnancies with SLE, but many of the outcomes are discussed in relation to SLE disease activity and treatments during pregnancy. In a retrospective study of 267 SLE patients, high levels of anti-dsDNA antibodies and hypocomplementemia during the second trimester were associated with poor pregnancy outcomes such as fetal death and preterm birth in the pregnant patients with residual SLE disease activity [6]. In the patients with APS, hypocomplementaemia has been reported to be associated with poor pregnancy outcomes, including preterm birth [16]. In the present study, because preterm birth occurred frequently in the APS patients with hypocomplementaemia, APS was not an independent factor for preterm birth, but attention may need to be paid to preterm birth in SLE patients with APS and hypocomplementemia.

Currently, there are limited reports on the management and treatment of SLE before pregnancy, which is important from the perspective of preconception care [4]. The present study showed for the first time that a pre-pregnancy serum C3 level < 85 mg/dl, in addition to a history of lupus nephritis, is a risk factor for preterm birth. Furthermore, the result that the preterm birth rate increases in pregnancy with low serum C3 level (< 85 mg/dl), even with SLEDAI ≤ 4, suggests that pre-pregnancy C3 level may be more useful than SLEDAI for predicting preterm birth. The target value of a serum C3 level > 85 mg/dl is higher than the general treatment standard for non-pregnancy (serum C3 levels of 70–75 mg/dl) in SLE patients. This result shows that pregnancy is a special state in SLE that can exacerbate its condition, and it is necessary to keep the disease activity of pregnant SLE patients more stable than that in non-pregnant patients to avoid preterm birth. In addition, SLE patients with a history of lupus nephritis have an increased risk of preterm birth and may require strict control of disease activity based on serum C3 levels. The present study showed that in pregnancy with SLE, it is important to perform preconception care focusing on organ lesions such as lupus nephritis and serum C3 levels before pregnancy to improve the pregnancy outcome, even if there is no activity such as SLEDAI ≤ 4.

Lupus nephritis and its history are associated with poor fetal outcomes and are also known as the risk of preterm birth in pregnancy with SLE [3]. This study also showed that a history of lupus nephritis is a risk of preterm birth. This result supports previous reports and indicates that we need to give special emphasis to preconception care for SLE patients with a history of lupus nephritis.

In pregnancy with SLE, it is necessary to pay attention to exacerbation of SLE disease activity and, at the same time, management of the mother. Pregnancy is known to trigger exacerbations of SLE [17]. So far, high disease activity during pregnancy, severe renal impairment, pulmonary hypertension, and central nervous system disorders have been reported as risk factors for SLE relapse [18]. The requirement for permitting pregnancy in SLE patients is generally low disease activity or remission during the 6 months prior to pregnancy [3]. Even in cases in which the disease activity of SLE is stable, SLE can worsen during pregnancy in one-fourth of women and cause preterm birth in one-third of them [4]. Furthermore, even in women with SLEDAI < 4 before pregnancy, the incidences of SLE flare and pregnancy complications are higher than those in healthy subjects [4, 5]. The present study showed no difference in SLEDAI scores between the preterm and full-term birth groups from pre-pregnancy through the entire gestation period. However, the serum C3 levels in the preterm birth group were lower than those in the full-term birth group from before pregnancy through the entire pregnancy. Elevated serum complement levels are a phenomenon seen in normal pregnancies, but the pre-pregnancy rate of complement elevation was lower in the low C3 group (< 85 mg/dl) than that in the high C3 group (≥ 85 mg/dl). In the present study, there were many women in whom the activity of SLE was well controlled, so this could be a possible reason for no difference in the SLEDAI score between the preterm and full-term birth groups. However, the serum C3 levels were different between the two groups, suggesting that changes in serum C3 levels may sharply reflect disease activity in pregnancies complicated by SLE.

This study has some limitations. First, it was performed with a small number of patients at a single institution. However, this enabled us to perform the study with unified criteria for monitoring the data of each patient. Second, the type of lupus nephritis could not be identified by renal biopsy in some patients, and therefore, evaluation of the outcome of pregnancy based upon disease type was not possible. Third, changes in the types and usage of immunosuppressants in the pregnant patients with SLE over time may have affected study outcomes. Moreover, although the outcome of pregnancy and SLE disease activity in the mothers could be evaluated up to 1 year after delivery, subsequent growth of the children or long-term outcomes of the mothers could not be evaluated.

## Conclusion

In preconception care of pregnancies in women with SLE, it is important to reduce the risk of obstetric complications such as preterm birth and improve the prognosis of the mothers and children by fully controlling disease activity and strict pregnancy planning. The present study showed that management focusing on the history of lupus nephritis in addition to serum C3 levels in preconception care is important for predicting and preventing preterm birth in women with SLE. Prediction of preterm birth stratified by these risk factors will help rheumatologists and obstetricians who are struggling to manage pregnancies with SLE. In addition, this preconception care may increase the safety of the life events of pregnancy and childbirth, which have been a challenge for SLE patients. We hope that further research will enable SLE patients to become pregnant, give birth safely, and enrich their lives by spreading preconception care to all pregnant women with SLE.

## Data Availability

The data underlying this article will be shared on reasonable request to the corresponding author.

## Abbreviations

SLE: Systemic lupus erythematosus
C3: Serum complement 3
SLEDAI: SLE Disease Activity Index
LLDAS: Lupus low disease activity state
APS: Antiphospholipid syndrome
GC: Glucocorticoids
IVCY: Intravenous cyclophosphamide therapy
AZA: Azathioprine
TAC: Tacrolimus
HCQ: Hydroxychloroquine
LDA: Low-dose aspirin
LMWH: Low molecular weight heparin
anti-dsDNA Ab: Anti-double-stranded DNA antibodies
anti-Ro/SS-Ab: Anti-Ro/SSA antibodies
LA: Lupus anticoagulant
aCL: Anticardiolipin
Β2Gp1: Anti-β2-glycoprotein 1
anti-RNP Ab: Anti-RNP antibodies
anti-Sm Ab: Anti-Sm antibodies
